# The lived experience of patients going under hyperbaric oxygen therapy in Saudi Arabia: A phenomenological study

**DOI:** 10.1097/MD.0000000000038840

**Published:** 2024-07-26

**Authors:** Bayan Alilyyani, Nada Alaidarous, Manal Alsaedi, Sara Alshomrani, Shujun Aljuaid, Salha Alotaibi, Alanoud Alotaibi, Ghadah Alotaibi

**Affiliations:** a Department of Nursing Management and Education, College of Nursing, Taif University, Taif, Saudi Arabia; b Arthur Labatt Family School of Nursing, Western University, London, Canada.

**Keywords:** experience, hyperbaric, oxygen, patient, phenomenology, therapy

## Abstract

Hyperbaric oxygen therapy (HBOT) has emerged as an effective treatment or adjunct therapy for various disorders, prioritizing improving oxygen delivery to tissues. This qualitative inquiry aims to explore the psychological experiences of patients undergoing their first hyperbaric session, focusing on the emotional responses and information needs of patients. The study addresses the gap in understanding patient experiences and seeks to contribute to holistic care approaches and improved health outcomes for patients undergoing HBOT. Using a phenomenological-hermeneutical approach, this study engaged 6 participants from KAASH in Taif City who had undergone at least 1 HBOT session. Data were collected through in-person interviews using a semistructured guide, focusing on patients’ initial HBOT experiences. Giorgi 4-step method was applied for data analysis, allowing for rich descriptions and thematic categorization of the participants’ lived experiences. The analysis is centered around “Walking into the unknown” with emerged subthemes (feeling anxious or being calm; immediate or evolving outcomes; formal orientation and management; success stories and self-learning) organized under 2 revised categories, “Transitioning from fear to reassurance” and “Navigating the path of understanding.” The study also highlighted the critical role of healthcare providers in addressing information gaps and enhancing patient preparedness. The findings revealed the importance of comprehensive patient education and effective communication by healthcare providers to alleviate initial fears and improve treatment experiences for HBOT patients. Future research should expand to other regions and include healthcare providers’ perspectives to further validate these findings and enhance HBOT practices. Integrating psychological support into the treatment process may also benefit patients, promoting holistic care and improving overall health outcomes.

## 1. Introduction

Hyperbaric oxygen therapy (HBOT) is a treatment method that has acquired significant attention in the medical community due to its mechanism of action and healing qualities. HBOT can be prescribed for elective or emergency medical conditions as a primary or supportive therapy that involves delivering high concentration of oxygen at increased atmospheric pressure within a closed chamber.^[1–3]^ Over the years, HBOT has been studied extensively for its effects on patients with diverse health issues, including carbon monoxide poisoning, gas gangrene, nonhealing wounds (such as thermal burns, diabetic foot ulcers, and traumatic brain injuries),^[2,3]^ and hypoxia in coronavirus disease 2019 patients.^[3]^ While some clinicians suggest the use of HBOT in treating various inflammatory and systemic disorders, such as cerebral palsy, multiple sclerosis, or stroke, there is little evidence backing those claims.^[1,2]^

HBOT has shown significant improvement in overall health outcomes including limited side effects and long-term healing impacts. For example, in a recent systematic review concerning efficacy and safety of HBOT in treating patients with diabetic foot ulcers,^[3]^ studies shown a lowered risk of amputation and few, often mild, adverse effect, with middle ear barotrauma being the most common. More severe but rarer pressure-related complications, reported in lengthy treatment courses, include central nervous system oxygen toxicity, manifesting in self-limited and generalized seizures, or retinal abnormal changes linked to posttreatment, but also intertwined with diabetes poor prognosis.^[4]^ The literature suggests that alleviating risks and establishing efficacy of HBOT require attention to providing appropriate treatment and patient care.

The role of nurses in and around HBOT initiatives ranges widely from directly managing the program to overseeing or providing support (e.g., wound care, self-care, and patient education) during the treatment process. Within this role, establishing general safety measures is one of other key aspects in optimizing appropriate treatment delivery. Person-centered care is central to nursing knowledge and praxis, which involve research-based practice and vis-à-vis. Much has been written and promoted about best practices to ensure facility safety for patients undergoing HBOT; however, research suggests that attention to psychosocial factors shaping patient’s experience, like anxiety, stress, or compliance, is important to alleviate potential treatment risks, and consequently, improve health outcomes.^[5]^ Understandings of subjective experiences of patients’ undergoing HBOT often overlooked in the delivery of care.^[5,6]^ Attention to patients’ perspective on healthcare delivery helps limiting missed care opportunities to improve quality of care according to the principles of patient-centered care.

## 2. Context of HBOT applications

The therapeutic use of HBOT was first documented in 1873 when miners received the treatment for decompression sickness.^[2]^ Toward the late 20th century, guidelines for using HBOT have improved due to rigorous experimentation and clinical trials. In 2011, HBOT became a recognized effective treatment for 14 conditions, including gas gangrene, embolism, acute ischemic wounds, skin grafts, brain abscess, radiation burn, and acute unilateral hearing loss.^[2]^ Most of these conditions require completing a treatment course that involves a series of sessions delivered over a period and under specific depending on each patient’s condition.^[3]^

In Saudi Arabia, the application of HBOT began in 1979 when the first pressure room for oxygen therapy was established at the Armed Forces Hospital Abdulaziz Naval Base in the Eastern Region. Since then, it has become a central department in the country, with 3 chambers for HBOT, dedicated to patients with hypoxic conditions and nonhealing wounds.^[6]^ In 2014, the Health Outreach Services Department declared HBOT a National Program, focusing on developing treatment quality and clinical standards that are unified across the country and in accordance with the efficacy and safety guidelines governing hyperbaric medicine. Although the potential benefits of this treatment method and its appropriate practice have been recognized, the availability of hyperbaric treatment facilities in Saudi Arabia remains limited.^[7]^

Moreover, the role of nurses in and around HBOT application ranges widely from direct management or oversight (e.g., program director or nurse manager) to bedside care (e.g., wound care, pain assessment, and patient education) during the treatment delivery. Within this role, establishing general safety measures is one of the other key aspects of optimizing appropriate treatment delivery. Holistic care is central to nursing knowledge and praxis, which involves research-based practice and vis-à-vis. Much has been written and promoted about best practices to ensure facility safety for patients undergoing HBOT; however, research suggests that attention to psychosocial factors shaping patient’s experience, like anxiety, stress, or compliance, is important to alleviate potential treatment risks, and consequently, improve health outcomes.^[5]^

## 3. Overview of psychological experiences and HBOT

Various studies have explored the psychological effect of HBOT on health outcomes. For instance, Feng and Li^[8]^ conducted research in China to investigate how HBOT impacts patients with incomplete spinal cord injuries regarding psychosocial issues and nerve function, specifically related to depression and anxiety. After 8 weeks of treatment, both the HBOT and psychotherapy groups demonstrated significantly lower Hamilton depression rating scale scores compared to the control group. Another interesting research conducted by Harch et al^[9]^ at Louisiana State University School of Medicine, New Orleans, assessed the safety, feasibility, and effectiveness of HBOT for mild traumatic brain injury and posttraumatic stress disorder in 29 military veterans with blast-induced concussions. After 40 sessions of HBOT, the veterans exhibited improved performance in physical, psychological, and cognitive tests. Notably, those who received HBOT showed enhancements in memory, attention, anxiety, and depression, including reduced suicidal thoughts, leading to a decreased reliance on psychoactive medications.^[9]^

Furthermore, Harch and Fogarty^[10]^ conducted an observational case report study to examine the effectiveness of HBOT on Alzheimer disease. The study focused on a 58-year-old woman whose cognitive function had deteriorated for 5 years. After undergoing 40 sessions of HBOT, she reported improved moods, energy levels, and daily task performance after just 21 sessions. She also experienced enhancement in concentration, memory, sleep, and computer ability after completing the 40 HBOT sessions.^[10]^ Similar outcomes were observed in a recent qualitative descriptive study conducted in an HBOT clinic in Istanbul aimed to assess the anxiety levels of patients undergoing their first HBOT treatment. The researchers highlighted that the participating patients reported high anxiety levels upon their arrival at the clinic, which significantly decreased after the HBOT treatment.^[11]^ These valuable insights into the psychological effects of HBOT could potentially demonstrate its benefits in improving health outcomes related to injuries, posttraumatic stress disorder, mood, and sleep disorders. Studies also provide insights into patients’ concerns that could be overcome once they start the treatment.

The patient experiences of HBOT have been explored through several qualitative studies, appear to be diverse and can vary based on the specific setting, patient population, and the context of the treatment. Baines et al^[12]^ conducted a study in a tertiary hospital in Australia, aiming to improve the patient experience by monitoring blood glucose and lowering anxiety caused by low blood glucose. The research demonstrated that HBOT positively affected patients but occasionally raised concerns about dropping blood sugar levels. Another Australian study was conducted in a hyperbaric center by Chalmers et al,^[13]^ examining patients’ past knowledge and experiences of HBOT. The results highlighted several problems faced by patients during the treatment, including uncertainty about the treatment, discomfort arising from chamber noise and coldness, and difficulties related to wearing masks or hoods. Clearly, these challenges impacted patients’ comfort and satisfaction during HBOT sessions, influencing their overall experience with the therapy.

In addition, Carvalho Machado et al^[5]^ conducted qualitative research in Brazil focusing on the emotional responses of HBOT patients. This study found that patients undergoing HBOT reported experiencing a range of emotions, including fear, worry, exhaustion, happiness, and hope. Similarly, a qualitative study by Velure et al^[14]^ in Norway investigated the treatment experiences of patients with pelvic cancer undergoing HBOT. Patients in this study expressed feelings of anxiety and worry before the treatment, which is understandable given their medical condition and the unfamiliarity with the HBOT process. These emotional responses indicate that the treatment can evoke various feelings in patients, possibly influenced by factors such as treatment effectiveness, side effects, and overall well-being during and after the sessions.

## 4. Significance of current study

This qualitative inquiry aims to explore the often overlooked yet important aspect of treatment outcomes, the psychological experiences of patients undergoing HBOT. The first HBOT session is a significant milestone for patients, often evoking a mix of emotions including hope, anxiety, and fear, highlights the importance of addressing associated needs beyond treatment administration.^[5,6]^ Despite advancements in understanding the role of HBOT in treating health issues,^[1,2]^ there remains a notable knowledge gap concerning patient experiences across different contexts and their potential to inform appropriate treatment and relevant practices.^[5,7]^ This phenomenological study aimed to bridge this gap by exploring patients’ experiences undergoing their first HBOT in Saudi Arabia, particularly in Taif City, where such research is lacking. The overarching goal of this research is contributing to the promotion of holistic care approaches, appropriate treatment delivery, and improved health outcomes among targeted population (i.e., patients undergoing their first HBOT) locally and across similar contexts.

## 5. Material and methods

### 5.1. Design

**Figure 1.**
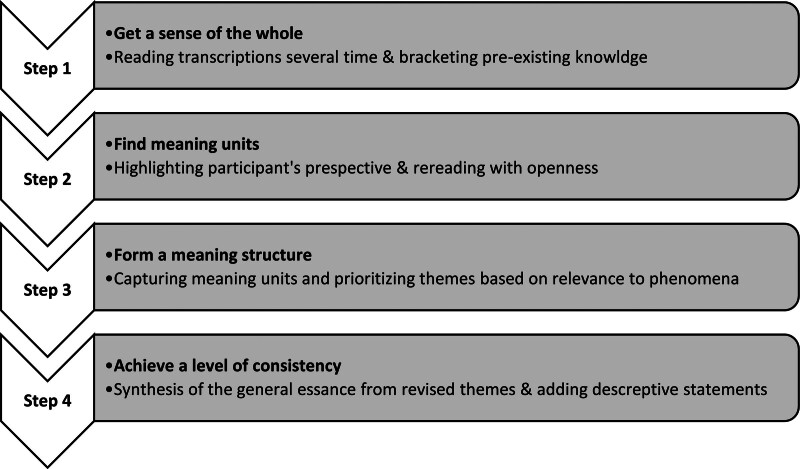
Giorgi 4-phase phenomenological method for meaning analysis.

This qualitative study employed a phenomenological-hermeneutical approach^[14]^ to explore the lived experiences of patients receiving HBOT and their interpretations. Aiming to provide rich descriptions of participants’ experiences, this design encourages researchers to engage in a hermeneutical process of interpretation (i.e., considering the researchers’ pre-knowledge/perceptions and the broader context of lived experiences); and an iterative process (including ongoing dialogue with participants, research team debriefings, and relevant literature). Giorgi 4-step method^[15]^ was applied to determine the subjective meaning of participants’ experiences and while achieving a degree of objectivity (Fig. 1).

This method was chosen given its alignment with descriptive phenomenological tradition; phenomena description that centered around discovery rather than verification; techniques to describe the structure of the experience that focus on each participant’s perspective without abstracting their viewpoint out through analysis (e.g., bracketing pre-knowledge on the effects of HBOT). The phenomenological approach also facilitates the selection of a small number of participants with unique and intersecting experiences, enabling comprehensive exploration and in-depth analysis of subjective meanings.^[16]^ A purposeful sampling method was employed to recruit patients who are able to answer the research question: “What are the lived experiences of patients undergoing their first HBOT in the outpatient department?”

### 5.2. Participants

The participants were undergoing HBOT for different conditions, including poor wound healing following surgery and/or radiotherapy, with diagnoses encompassing hearing impairment, nonhealing ulcers, radiation necrosis, and wound breakdown in avascular areas. Six participants were recruited from the outpatient department (OPD) at King Abdulaziz Specialist Hospital, which was the first facility in Taif City to establish HBOT chambers.

The participants in this study had undergone at least 1 HBOT, were willing to talk about their first hyperbaric experience, and had a minimum age of 18. Patients who were severely ill or unable to communicate verbally were excluded from the recruitment. The participants’ ages spanned a wide range from 23 to 72, with a mean age of 52. The reported diagnosis for most participants was a diabetic foot followed by hearing loss reported by 2 participants (female and male); detailed descriptions are illustrated in Table 1.

**Table 1:** Description of participants.

Participants	Age	Gender	Number of prescribed HBOT sessions	Diagnosis
1	72	Male	17	Diabetic foot
2	67	Male	16	Diabetic foot
3	26	Female	14	Hearing loss
4	23	Male	19	Hearing loss
5	60	Male	2	Diabetic foot
6	65	Male	4	Diabetic foot

HBOT = hyperbaric oxygen therapy.

### 5.3. Ethical considerations

Ethical approval for the study was obtained from Health Affairs in Taif (IPR NO: 763, Date: December 23, 2022). Subsequently, permission from leadership in the selected hospital was also obtained. During the recruitment phase, eligible participants were identified. After the first author or the OPD-assigned physician explained the nature and purpose of the study and the informed consent was obtained from each interested participants, the researchers scheduled the individual interviews. During each interview, the eligible participants were reminded of the research purpose, data privacy and confidentiality procedures, and their right to withdraw from the study. A copy of the information letter and signed consent was given to each participant.

### 5.4. Data collection

The researchers collected data from eligible participants by conducting in-person interviews and field notes at the OPD’s meeting room from January to July 2023, with the interview date and time arranged according to the participants’ convenience. All interviews were conducted in Arabic and audio-recorded for transcription and analysis purposes. Field notes of verbal and nonverbal cues were also documented during or directly after the interview.

The researchers used a semistructured interview guide with open-ended questions to allow the participants to tell as much as possible about the experiences related to HBOT. The key statement was “Please, tell me about your first therapy session?” Additional questions were used, depending on how much the participant felt comfortable sharing. Some examples of questions are presented in Table 2.

**Table 2:** Example of interview questions used in 1 interview.

**Primary research question:** *What are the lived experiences of patients undergoing their first hyperbaric oxygen therapy in the OPD?*
Please, tell me about your first therapy session. How did you feel about it? What do you mean by “the treatment felt imposed?” What did you expect to happen? What do you think you should have known before starting the therapy? Have you noticed any different feelings during the session? Can you tell me more about the “feeling of pressure?” How did you feel after completing the session? How do you explain it? What are the things that have helped you during this experience?

OPD = outpatient department.

Each individual interview lasted from 30 to 60 minutes and the interviews were transcribed by a professional transcriber fluent in Arabic. The complete collection of interviews consisted of 285 transcribed pages, ranging from 15 to 35 pages per participant. The transcriptions were discussed with the research team in debrief meetings and were not returned to the participants for comments. The research team was meeting regularly to maintain coherence across interviews, ensure topic areas were covered, and encourage the iterative process (reflecting, questioning, and debriefing) between the research team. For example, there was only 1 female eligible participant participated in this study; during the team debrief, some questions were raised concerning HBOT information prior sessions, and a follow-up interview was scheduled with the participant to explore further meanings and interpretations.

### 5.5. Data analysis

The researchers applied Giorgi descriptive phenomenology 4-step method to analyze the collected data while ensuring rigor and validity at every step^[15,17,18]^ which is briefly presented in Figure 1. In preparation for data analysis, all transcripts and field notes were converted into an electronic format to facilitate collaborative coding.^[16]^

#### 5.5.1. Step 1

The researchers immersed themselves in reading each transcribed interview and field notes several times, gaining a deep understanding of the participants’ accounts as a whole.^[15]^ A general description of each participant’s experience of undergoing their first HBOT was produced along with reflections, which were discussed with the research team to engage in the process of awareness (e.g., bracketing researchers’ preexisting knowledge or experience) and openness (e.g., looking at participants’ accounts in a fresh way).^[14,17,18]^

#### 5.5.2. Step 2

The transcripts were discerned into sections based on transitions in meaning of experiences (including accounts/stories, feelings, responses, or expectations) from each transcription, using participant’s words or expressions to find explicit meanings.^[15,17]^ The researchers noted every time a participant referred to response, outcome, or information related to their first hyperbaric experience.

#### 5.5.3. Step 3

**Figure 2.**
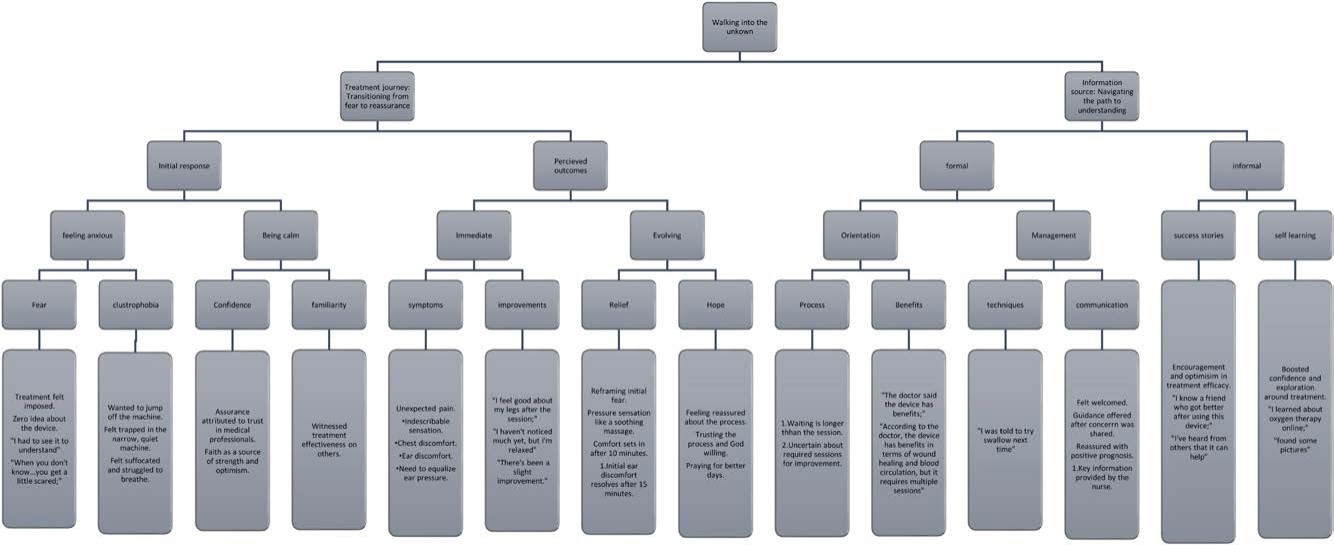
The eidetic structure of subjective meaning units on “Walking into the unknown.” It presents how the essential theme, and the subthemes were constructed from the meaning units. The hierarchy label represents the value of the theme; arranged from the most valuable, from participant’s perspective.

The identified meaning units (i.e., few words to entire sentence) were translated to English, with attention to reproducing general and rich meaning rather than literal, to facilitate a third-person perspective.^[15,17]^ Two researchers (BA and NA) reviewed each transcript, refined the translated meaning units identified by the other researchers, and then performed back-translation to ensure the clarity and accuracy of the subjective meaning units highlighted in the original language in the text. Each subjective meaning unit that the participant explicitly used to describe, for example, the experiences of fear before commencing therapy sessions, were identified then carefully coded in English and restructured into a descriptive heading (or theme). The descriptive headings, linked with the corresponding original texts, were arranged from the most valuable, from participant’s perspective, then clustered depending on how the notion of the headings (i.e., emerging concepts or ideas) relate to each other. Figure 2 presents examples of the meaning analysis of walking into the unknown. The researchers noted connections between the preexisting notions and the explicit descriptions made by participants, embracing the intersubjective relationship in between the researcher and researched.^[18]^

#### 5.5.4. Step 4

Themes about patients’ experience of undergoing first HBOT were derived by articulating the broad meaning units from all participants, combining and/or renaming headings (or themes), and providing a comprehensive, fresh, and complex understanding of the data, following the phenomenological tradition.^[15,18]^

The initial synthesis was produced by the research team under the direction of the first author (BA); the final synthesis was performed by the second author (NA). The study had an adequate number of participants who could answer the research question as no new meaning units were found after the sixth participant’s interview, indicating data saturation.

## 6. Results

The analysis is centered around “Walking into the unknown” with emerged subthemes (feeling anxious or being calm; immediate or evolving outcomes; formal orientation and management; success stories and self-learning) organized under 2 revised categories, “Transitioning from fear to reassurance” and “Navigating the path of understanding.” Figure 2 presents the eidetic structure of meaning analysis of “Walking into the unknown” and the relationship between the subthemes. The most valuable subtheme, “feeling anxious or being calm,” reflects the initial emotional responses of patients undergoing their first HBOT. Determining the value of the subthemes was possible using recurring ideas or notions among the multiple descriptions of the experience of the included participants.

### 6.1. Transitioning from fear to reassurance

Two primary aspects emerged through the analysis of participants’ psychological experiences with HBOT: initial fear or anxiety, and the eventual reassurance and perceived outcomes.

#### 6.1.1. Initial fear and anxiety

The participants expressed initial fear or anxiety before their first HBOT session, primarily due to a lack of understanding of what the treatment involved. As 1 patient mentioned, “*In the beginning, when you don’t know anything about it, right? So, you might be a little scared*” (patient 5). This feeling was echoed by other participants; the uncertainty about how their body would react to the treatment and whether it would be effective added to their apprehension. The participants expressed initial fear or anxiety before their first HBOT session, primarily due to a lack of understanding of what the treatment involved. As 1 patient mentioned, “*In the beginning, when you don’t know anything about it, right? So, you might be a little scared*” (patient 5). This feeling was echoed by other participants; the uncertainty about how their body would react to the treatment and whether it would be effective added to their apprehension. Patient 1 shared their initial unease: “*On the first day, I felt like I wanted to jump off the machine because it’s a narrow and quiet place. I felt like I was suffocating and couldn’t breathe properly and had palpitations, but I don’t know if it was from the machine or from fear*.”

#### 6.1.2. Diminishing fear and discomfort

However, several participants noted that these feelings of fear diminished after the first session. Patient 5 mentioned, “*It was only during the first session, and then it was normal*,” and patient 6 concurred, stating, “*Fear or distress did not last and was just at the beginning of the first session*.” Other participants expressed being even more relaxed inside the machine as time goes by, for example: patient 5 shared: “*After about 20 min, I feel like there’s pressure on me from the capsule inside, but it’s a good feeling and it reaches the body like some kind of massage, you feel like the body is relaxed, not just feeling it*.” Participant 2 also shared a similar sentiment: “*In the device, I feel better and reassured*,” indicating a positive response during the treatment.

#### 6.1.3. Noticing effects and improvements

The participants’ experiences with the perceived outcomes of HBOT varied, with many noting manageable side effects and some immediate improvements. While barotrauma was a common issue, it was easily managed by techniques such as swallowing to equalize inner ear pressure. Patient 1 vividly described their experience: “*I definitely felt some pain. I couldn’t even describe it; now, I don’t feel it anymore. Also, there’s some discomfort in the ear at a certain stage, after 15 minutes, but then things settle down... before leaving I asked the nurse if that was normal and she said I should try swallowing next time*.”

The experiences varied among patients, reflecting individual differences in health conditions and their responses. Patient 4 felt a modest improvement, noting, “*maybe 10% improvement*,” while patient 3 was uncertain about the benefits but remained hopeful, “*God willing, I feel good*.” Despite the lack of perceived improvements, patients were hopeful about the therapy’s potential benefits. Patient 1 mentioned a slight improvement, stating, “*Honestly, until now, I haven’t really felt anything, you know? There was a slight improvement*.”

#### 6.1.4. Emerging relief and hope

Despite initial discomfort, many patients reported noticeable improvements in their condition shortly after beginning HBOT. For instance, patient 4 expressed, “*I feel good now about my legs after the session*,” while patient 6 was informed by the doctor about their healing progress, “*Today, the doctor told me my wound healing process is good*.” This immediate improvement, especially in pain relief and wound healing, was a clear motivator for patients to feel hopeful reassured about continuing the therapy. Almost all patients were vocal about their trust in therapy, represented by hope regardless of improvement, and grateful to God for the treatment availability and noticeable improvement in their health condition, as expressed by patient 5: “*Thank God, I felt nothing in my chest, and it has improved a bit, and my condition has improved*.”

#### 6.1.5. Addressing concerns and building trust

While sharing their positive experiences, some participants expressed concerns about the observed improvements in their condition might be temporary, highlighting the importance of effective communication and trust between doctors and patients to address and clarify any anxieties or uncertainties they may have with HBOT treatment. This was evident in the experience of patient 6, who mentioned, “*At the beginning, when you don’t know anything about it, you might be a little scared. After that, you just spend 10 minutes in the machine, and then you get comfortable*.” The participants also pointed out that some HBOT chamber healthcare team promptly addressed their voiced concerns, contributing to their positive outlook on the treatment journey.

### 6.2. Navigating the path to understanding

#### 6.2.1. Uncertainty and lack of information

The analyzed interviews highlight a concerning trend among the participants regarding the notions of uncertainty and insufficient information about HBOT and its associated side effects. When asked about receiving information on or having prior knowledge of how the therapy, it appeared that many patients entered the therapy without a clear understanding of how the device worked or what to expect during and after the sessions. One participant, patient 1, responded: “*To be honest, I didn’t have any information about it.*” This statement reflects a shared sentiment by other participants, expressing that they were essentially uninformed about the therapy before starting it. Another participant, P2, emphasized the need for more support and comprehensive information from the healthcare providers, candidly expressing, “*I need more information. According to the doctor, the device has benefits in terms of wound healing and body activation, but it requires multiple sessions, like two, three, four, or five, after many sessions. That is just what the doctor told me.*”

#### 6.2.2. Unpreparedness despite information

Despite receiving written information about the treatment protocol from the HBOT unit beforehand, all participants expressed difficulty in understanding the actual experience of undergoing HBOT. They felt unprepared and uncertain about what to anticipate during the treatment. Patient 5 highlighted, “*I need more information... the picture was zero in terms of the device*.”

#### 6.2.3. Moving through despite uncertainty

Despite the observed high levels of unfamiliarity and uncertainty surrounding HBOT, the participants still took the chance to begin the treatment, as expressed by patient 3: “*The picture was unclear in terms of the device; it was just something imposed on me as a treatment*.” This willingness to proceed despite doubts indicates a level of trust in the treatment’s potential benefits.

#### 6.2.4. Exploring and self-educating

Interestingly, some patients had the technical capacity to self-learning through online sources to fill the information gap left by healthcare providers. The experience of patient 4 exemplifies this, “*I learned about oxygen online and found some pictures.*” This proactive approach to self-education suggests that patients seek knowledge to make informed decisions about their treatment. Others expressed a desire for more comprehensive explanations about the therapy. The need for better communication and thorough information sharing from healthcare providers is a recurring subtheme, highlighting the importance of clear and detailed patient education in HBOT applications.

## 7. Discussion

The study findings indicate that participating patients undergoing HBOT received insufficient information about the treatment, highlighting the importance of healthcare providers’ role in promoting active and relevant approaches to prepare patients for HBOT. The lack of adequate information provided by healthcare providers seems to create a sense of uncertainty and confusion among patients. Patient 1 highlighted this issue, stating, “To be honest, I didn’t have any information about it.”

These findings align with other research that encourages healthcare professionals to proactively educate patients, equipping them with the necessary knowledge about the treatment and its related distress in advance.^[14]^ To address the information gaps, building capacity among healthcare providers to ensure that patients are well-informed and better prepared to undergo the treatment is key, ultimately leading to improved treatment experiences and overall health outcomes.

Moreover, the study’s findings showed that patients reported noticeable improvements in their conditions following the treatment, highlighting the potential effectiveness of the treatment and its impact on patients’ overall health and well-being. Patient 4 expressed this sentiment, stating, “I feel good now about my legs after the session.” These findings corroborated earlier research indicating that participants experienced improvements in memory, attention, anxiety, and depression following their HBOT sessions.^[9]^ Furthermore, in a study conducted by Harch and Fogarty^[10]^ that explored the efficacy of HBOT in Alzheimer disease, a patient observed enhancements in both memory and moods after undergoing 40 sessions of HBOT. The significant impact of HBOT on disease treatment was evident, with all patients in this study reporting some sort improvements in their condition and general well-being, even after 1 session.

Some patients in this study reported experiencing fear and apprehension related to the HBOT treatment or the device itself, although these feelings tended to diminish after their first session. Patient 5 shared, “It was only during the first session, and then it was normal.” This observation aligns with findings from other studies, which highlight that patients often feel worried and anxious, particularly before starting the first treatment session, was highlighted across different populations.^[5,14]^ The findings of this study also align with a recent study that observed a noticeable decrease in fear and anxiety immediately after the initial session.^[11]^ While HBOT offers numerous benefits, as indicated by patients in this study and previous research,^[9,12,14]^ it is essential to acknowledge that fear and anxiety that can accompany this treatment method.

### 7.1. Strengths and limitations

A major strength of this study is that, as far as it is known, it is the first study conducted in a clinical setting in Saudi Arabia to capture the lived experiences of patients undergoing HBOT. The analysis of participants’ interpretation followed a transparent process informed by the selected methodological approach to ensure rigor and objectivity in navigating the various meanings expressed by participants, validated by descriptive quotations. This qualitative approach heavily relies on participants’ subjective experiences and narratives, indicating potential biases, as participants may remember or interpret events differently based on their perspectives.^[17]^ Moreover, the research team debriefings were centered around promoting aspects of rigor and internal validity at each step in this research, ensuring hermeneutic reflection and intersubjectivity.^[18]^ Within the sample, HBOT was generally considered an effective treatment with minimal side effects; however, it is important to note that these unique experiences may limit the applicability of the findings beyond the group understudied.

### 7.2. Recommendations for future studies

As the first study exploring the experience of patients receiving HBOT in Saudi Arabia, there is a clear need for further research in the country. Future studies should employ both qualitative and quantitative designs to investigate the effects of HBOT comprehensively. Expanding research to other regions of Saudi Arabia would enhance the understanding of this treatment’s impact across diverse populations. Additionally, future research should consider incorporating healthcare providers’ perspectives in the context of patients receiving HBOT.

### 7.3. Implications for practice

To ensure and promote holistic care for patients, it is crucial that healthcare providers enhance their understanding and knowledge in caring for patients undergoing HBOT. The findings indicate the importance of providing information in advance, regularly monitoring treatment progress after each session, and transparently communicating it to patients. Additionally, patients should openly discuss any concerns or side effects with their healthcare providers. Since psychological aspects were the most frequently reported by patients, it suggests that integrating a psychologist into the treatment process may benefit individuals undergoing HBOT.

## 8. Conclusion

HBOT was perceived as stepping into the unknown by several patients, and they expressed the need for comprehensive information to alleviate distress and anxiety before starting the treatment. Further research with larger and more diverse samples is essential to validate these findings and establish a stronger evidence base for the efficacy of the treatment. Attention to patients’ perspective on healthcare delivery helps limit missed care opportunities and improve quality of care in accordance with the principles of holistic care and safe delivery in HBOT.

## Acknowledgments

The authors like to acknowledge the Deanship of Scientific Research, Taif University for funding this work.

## Author contributions

**Conceptualization:** Bayan Alilyyani, Manal Alsaedi, Sara Alshomrani, Shujun Aljuaid, Salha Alotaibi, Alanoud Alotaibi, Ghadah Alotaibi.

**Formal analysis:** Bayan Alilyyani, Manal Alsaedi, Sara Alshomrani, Shujun Aljuaid, Salha Alotaibi, Alanoud Alotaibi, Ghadah Alotaibi, Nada Alaidarous.

**Investigation:** Bayan Alilyyani, Nada Alaidarous.

**Methodology:** Bayan Alilyyani, Nada Alaidarous.

**Project administration:** Bayan Alilyyani, Nada Alaidarous.

**Supervision:** Bayan Alilyyani, Nada Alaidarous.

**Validation:** Bayan Alilyyani.

**Visualization:** Bayan Alilyyani.

**Writing—review & editing:** Bayan Alilyyani, Nada Alaidarous.

**Data curation:** Manal Alsaedi, Sara Alshomrani, Shujun Aljuaid, Salha Alotaibi, Alanoud Alotaibi, Ghadah Alotaibi.

**Resources:** Manal Alsaedi, Sara Alshomrani, Shujun Aljuaid, Salha Alotaibi, Alanoud Alotaibi, Ghadah Alotaibi.

**Software:** Manal Alsaedi, Sara Alshomrani, Shujun Aljuaid, Salha Alotaibi, Alanoud Alotaibi, Ghadah Alotaibi.

**Writing—original draft:** Manal Alsaedi, Sara Alshomrani, Shujun Aljuaid, Salha Alotaibi, Alanoud Alotaibi, Ghadah Alotaibi.
